# Research progress and design optimization of CAR‐T therapy for pancreatic ductal adenocarcinoma

**DOI:** 10.1002/cam4.2430

**Published:** 2019-07-03

**Authors:** Tianjiao Li, Hao Li, Shuo Li, Shuaishuai Xu, Wuhu Zhang, Heli Gao, Huaxiang Xu, Chuntao Wu, Wenquan Wang, Xianjun Yu, Liang Liu

**Affiliations:** ^1^ Department of Pancreatic Surgery, Shanghai Cancer Centre Fudan University Shanghai China; ^2^ Department of Oncology, Shanghai Medical College Fudan University Shanghai China; ^3^ Shanghai Pancreatic Cancer Institute Shanghai China; ^4^ Pancreatic Cancer Institute Fudan University Shanghai China

**Keywords:** CAR‐T, design optimization, immunotherapy, PDAC

## Abstract

Pancreatic ductal adenocarcinoma (PDAC) is a highly malignant cancer with limited treatment options. Chimeric antigen receptor T cells (CAR‐T) are genetically engineered T cells that can specifically kill tumor cells without major histocompatibility complex restriction. Encouraging progress in CAR‐T therapy for PDAC has been made in preclinical and early phase clinical trials. Challenges in CAR‐T therapy for solid tumors still exist, including immunosuppressive microenvironment, interstitial barrier, poor chemotaxis, and the “on‐target, off‐tumor” effect. Applying neoantigens of PDAC as targets for CAR‐T therapy, recognizing the CAR‐T subgroup with better antitumor effect, and designing a CAR‐T system targeting stroma of PDAC may contribute to develop a powerful CAR‐T therapy for PDAC in the future.

## 1 INTRODUCTION

Pancreatic ductal adenocarcinoma (PDAC) is highly malignant cancer with a 5‐year survival rate of less than 8%.^1^ Due to its increasing incidence, PDAC is expected to become the second leading cause of cancer death in the United States by 2030.^2^ An immunosuppressive microenvironment is an important factor that promotes the malignant phenotype of PDAC. Two groups of immune components with opposing functions in the PDAC microenvironment have been revealed—one group has positive immunoregulatory function and mainly includes CD4+ T lymphocytes, CD8+ T lymphocytes, and type‐1 macrophages (M1) and the Th1‐type cytokines secreted by these cells (such as IL‐2, IFN‐γ, TNF‐β, and IL‐12); the other group has a negative immunoregulatory function and mainly includes regulatory T cells (Treg), myeloid‐derived suppressor cells (MDSCs), and type‐2 macrophages (M2) and the Th2‐type cytokines (such as IL‐1, IL‐6, IL‐10, and TGF‐β), and immunological checkpoint regulators (such as PD‐1 and CTLA‐4) expressed by these cells.^3^ The negative immunoregulatory components have a greater impact than the positive immunoregulatory components in terms of content and function, which leads to an immunosuppressive microenvironment. PDAC cells can express chemokines (such as CCL2 and CCL5) and cytokines (such as GM‐CSF, VEGF, and PDGF) to induce the chemotaxis of inflammatory cells into the tumor stroma,^4^, ^5^ but only a small proportion of them are cytotoxic T lymphocytes (CTL) because of the mismatch of chemokine receptors on CTLs, the abnormal development of interstitial vessels, and the downregulation of endothelial adhesion molecules.^6^, ^7^, ^8^ In addition, cancer cells as well as Tregs, MDSCs, and M2 in the microenvironment can express Th2‐type cytokines and immune checkpoints, which can not only aid these cells in maintaining their immunosuppressive phenotype, but can also inhibit the antitumor effect of CD4+T/CD8+T cells,^9^ promote M1 to M2 transformation,^10^ and induce myelogenous cells to differentiate into MDSCs.^11^ Moreover, endothelial cells, cancer‐associated fibroblasts (CAF), and pancreatic stellate cells in PDAC stroma as well as hypoxia and metabolic reprogramming induced by strong interstitial reactions are all involved in the formation of an immunosuppressive microenvironment.^12^, ^13^ In addition, inhibitory immune cells not only mediate the immunological escape of pancreatic cancer cells but also promote their invasion, distant metastasis, stemness characteristics, epithelial‐mesenchymal transition (EMT), and angiogenesis.^14^, ^15^, ^16^ Therefore, reducing immunosuppression in the PDAC microenvironment or developing a new immune‐killing mechanism that targets PDAC cells may be a potential method of PDAC treatment. Therefore, chimeric antigen receptor T‐cell (CAR‐T) therapy has been studied and applied for the treatment of PDAC.

CAR‐T therapy was first developed for treating hematological malignancies and showed strong antitumor effects in clinical applications. CAR is a transmembrane protein that is expressed on T‐cell surface via biotechnology. CAR usually consists of three parts: (a) the extracellular segment, which is mainly composed of single‐chain fragment variable (scFv) derived from the immunoglobulin variable region and can specifically recognize tumor antigens; (b) the transmembrane segment, which is mainly composed of homologous or heterologous transmembrane regions from CD3, CD8, CD28, and FcεRI, anchors the CAR molecule in the cytoplasmic membrane and transmits the activation signal from scFv; (c) the intracellular segment, which consists of first signaling domain containing the immunoreceptor tyrosine‐based activation motif sequence and the second signaling domain from CD3ξ, 4‐1BB, and CD28. According to the number of second signaling domains contained in the intracellular segment, CARs are classified into three generations: the first generation (no second signal domain), the second generation (one second signal domain), and the third generation (two second signal domains). Various fourth and fifth generations of CARs have been developed to improve the performance and safety of CAR‐T therapy.^17^ The CAR enables CAR‐T cells to acquire specificity to tumor antigens, thus avoiding immune escape mediated by autoimmune tolerance. The CAR can provide the double activation signal needed for T‐cell activation. And, CAR‐T activation becomes more efficient without major histocompatibility complex (MHC) restriction, which avoids T‐cell dysfunction caused by the downregulation of MHC on the tumor surface. The excellent characteristics of CAR‐T cells demonstrate their great promise for treating solid tumors, including PDAC. Our review summarizes the target selection of CAR‐T for PDAC, the design optimization of CAR‐T therapy in solid tumors including PDAC, and the future development of CAR‐T therapy for PDAC.

## 2 CAR‐T TARGETS IN PDAC

### 2.1 Mesothelin

Mesothelin (MSLN) is the most widely studied target of CAR‐T therapy in PDAC. MSLN is highly expressed in many cancers, including pancreatic cancer, ovarian cancer, lung cancer, gastric cancer, colorectal cancer, and bladder cancer, while only a few MSLNs are expressed in mesothelial tissues in healthy people.^18^ The overexpression of MSLN on cancer cells presented in approximately 75%‐85% of patients with PDAC and is closely related to the postoperative recurrence and prognosis of PDAC patients.^19^ The high expression of MSLN in PDAC could activate the NF‐κB pathway and further induced the proliferation of cancer cells by autocrine or paracrine IL‐6 stimulation.^20^ Antiapoptotic proteins, Bcl‐XL and Mcl‐1, were upregulated in cancer cells with high expression of MSLN through the Akt/NF‐κB/IL‐6 pathway, which inhibited TNF‐α‐induced apoptosis.^21^ Moreover, binding to CA125/MUC‐16, MSLN selectively upregulated MMP‐7 secretion in PDAC cells via the p38 MAPK pathway, thus significantly enhancing the invasion and migration of PDAC cells.^22^ This evidence suggests that MSLN is an important molecule that promotes PDAC malignancy. In addition, recombinant immune endotoxin or vaccines targeting MSLN could effectively inhibit the proliferation, invasion, and metastasis of pancreatic cancer cells in vivo and in vitro, which further clarifies the significance of MSLN as a CAR‐T target in PDAC treatment.^23^, ^24^ He et al generated a second‐generation CAR‐T that targeted MSLN using the piggyBac transposon system. The MSLN‐CAR‐T could kill cancer cells with high MSLN expression in vitro.^25^ In a mouse model, approximately 30 days after 10^7^ CAR‐T cells were injected intravenously, metabolic imaging showed that tumors in these mice almost completely disappeared, while no other visceral dysfunction was observed. Similar results were also found in the study of Hua et al^26^ Currently, many clinical studies of MSLN‐CAR‐T therapy targeting PDAC are in progress. A phase I clinical trial involving six patients with advanced pancreatic cancer resistant to chemotherapy showed encouraging results. After CAR‐T treatment (three times a week for 3 weeks), two patients achieved progression‐free survival of 3.8 and 5.4 months.^27^ Metabolic activity (MAV) in tumors assessed by metabolic imaging (F18‐FDG) remained stable in three patients, while MAV of tumor decreased by 68.3% in another patient whose liver metastases completely disappeared. In addition, no cytokine storm effects or other CAR‐T‐related side effects were found in any of the patients.^27^

### 2.2 Carcinoembryonic antigen

Carcinoembryonic antigen (CEA) is highly expressed in approximately 65%‐75% of pancreatic cancers. High CEA expression is significantly associated with worse prognosis in PDAC. Knockout of the CEA family gene CEA‐related cell adhesion molecule (CEACAM) could significantly reduce the proliferation of cancer cells in vitro and increase the total survival time of mice bearing PDAC in vivo.^28^ Chmielewski et al^29^ designed a second‐generation CEA‐CAR‐T. In a mouse model with PDAC, the tumor treated with CEA‐CAR‐T cells shrank to the minimum detection limit, and the antitumor effect of CEA‐CAR‐T cells was not affected by serum CEA levels. Moreover, 45 days after tumor clearance by an injection of CEA‐CAR‐T cells, CEA (+) fibrosarcoma cells were subcutaneously transplanted, and the CEA‐CAR‐T cells still exhibited a powerful antitumor effect. To date, no clinical trials of CEA‐CAR‐T therapy targeting PDAC have been reported. A phase I clinical trial of CAR‐T therapy targeting CEA‐positive liver metastases (LM) from malignant tumors enrolled six patients, one of whom survived for 23 months with stable disease after treatment with a high dose of CAR‐T cells, and no serious CAR‐T‐related adverse events occurred. This result suggested that such CEA‐CAR‐T therapy has broad potential applications in patients with high tumor burden who have failed to respond to conventional therapy.^30^

### 2.3 Other targets of CAR‐T therapy for PDAC

In addition to the above targets, related studies used HER‐2, MUC‐1, NK‐R, PSCA, CD133, and CD24 as targets of CAR‐T therapy for PDAC^31^ (Table 1). The development of more specific targets will provide more options for the treatment of PDAC with CAR‐T therapy.

**Table 1:** Rarely studied targets of CAR‐T therapy for PDAC

Author	Target	Costimulatory molecule	Malignancy	Study types	Reference (PMID)
Golubovskaya	EGFR	GITR	Pancreatic and ovarian cancer	Mouse model	29772559
Golubovskaya	CD47	CD28	Ovarian, pancreatic, and cervical cancer	Mouse model	29065481
Whilding	Integrin‐αvβ6	CD28	Pancreatic and ovarian cancer	Mouse model	31091832
Posey	MUC1	4‐1BB	T cell leukemia and pancreatic cancer	Mouse model	27332733
Rataj	CD16	CD28	Pancreatic cancer, lymphoma, and melanoma	In vitro experiment	30429531
Hongwei Du	B7‐H3	CD28/4‐1BB	Pancreatic and ovarian cancer and neuroblastoma	Mouse model	30753824
Maliar	HER‐2/CD24	CD28	Pancreatic cancer	Mouse model	22819865
Abate‐Daga	PSCA	CD28/4‐1BB	Pancreatic cancer	Mouse model	24694017
Tal	NKp46	CD28/4‐1BB	Erythroleukemia, nonsmall cell lung cancer, cervix adenocarcinoma, and pancreatic cancer	Chick embryo chorioallantoic membrane model	25431955

## 3 PRINCIPAL CHALLENGES OF CAR‐T THERAPY FOR SOLID TUMORS INCLUDING PDAC

Several challenges limit the clinical appliance of CAR‐T therapy for solid tumors, including PDAC. Confronting these challenges and in‐depth study of their related mechanisms will contribute to a better CAR‐T treatment.

### 3.1 Challenges in side effects of CAR‐T therapy

The most common side effect of CAR‐T therapy is “On‐target, off‐tumor effect,” because tumor‐specific antigen was rarely identified as a target for CAR‐T therapy. Almost all CAR‐T therapy targets are tumor‐associated antigens (TAAs), which are often not completely foreign to the host, and activated CAR‐T cells may also harm healthy tissues expressing the same target. In a clinical trial of CAR‐T therapy against carboxy‐anhydrase‐IX (CAIX) in 12 patients with CAIX‐expressing metastatic renal cell carcinoma, four patients had increased level of liver enzymes corresponding to grades 3‐4 (CTC classification) after receiving the minimum dose of CAR‐T (2 × 10^8^ cells) and had to discontinue the trial. Liver biopsy revealed CAIX expression in the biliary epithelium and adjacent CAR‐T infiltration. Subsequently, after pretreatment with a CAIX monoclonal antibody, the four patients were treated with CAR‐T cells again without the recurrence of liver damage.^32^ Another side effect worth noting in CAR‐T treatment for solid tumors is cytokine release syndrome (CRS). Extensive CAR‐T activation lead to the oversecretion of cytokines that causes the CRS. In a study by Wang, mice with PDAC experienced anorexia and weight loss due to the increasing level of cytokines (IL‐6, TNF‐α, and IL‐1‐α), which was related to CAR‐T treatment.^33^ In current clinical practice, CRS is more common in CAR‐T therapy for hematologic tumors. CRS is sometimes observed in CAR‐T therapy of solid tumors and can usually be rescued by treatment with high dose of glucocorticoids and vasopressors, organ function support, and the IL‐6 receptor antibodies.^31^

### 3.2 Challenges from tumor microenvironment

What distinguishs solid tumors from hematological tumors is the abundant tumor stroma, which may explain that CAR‐T therapy is less effective in solid tumors than in hematological tumors. After CAR‐T cells are transfused into the circulation, CAR‐T for hematological malignancies would directly bind to malignant cells and initiate killing procedure, while CAR‐T for solid tumors have to infiltrate into the tumor stroma before exerting its antitumor effect. T‐cell homing to solid tumors is regulated by strict molecular mechanisms. Significant obstacles preventing CTLs homing have been clearly revealed, including microvascular dysplasia, abnormal expression of adhesion molecule, chemokine‐chemokine receptor mismatching, immunoediting expression of TAA, and recruitment of CAF.^6^ Immunosuppressive microenvironment is another important factor that inhibit the function of T cells, which have been mentioned above. CAR‐T cells, which are a type of CD4+/CD8+T cells, are also affected by the immunosuppressive network in the tumor microenvironment.^34^, ^35^ What's more, the biochemical characteristics of tumor stroma, including hypoxia, the lack of nutrition, and low pH, all disrupt the chemotaxis of CAR‐T cells and the resulting antitumor effects.^36^, ^37^ In addition, antigen modulation may occur after CAR‐T therapy due to the intratumoral heterogeneity, which also leads to CAR‐T dysfunction.^38^

## 4 OPTIMIZATION OF CAR‐T DESIGN

### 4.1 Improving targeting and specificity

**Figure 1:**
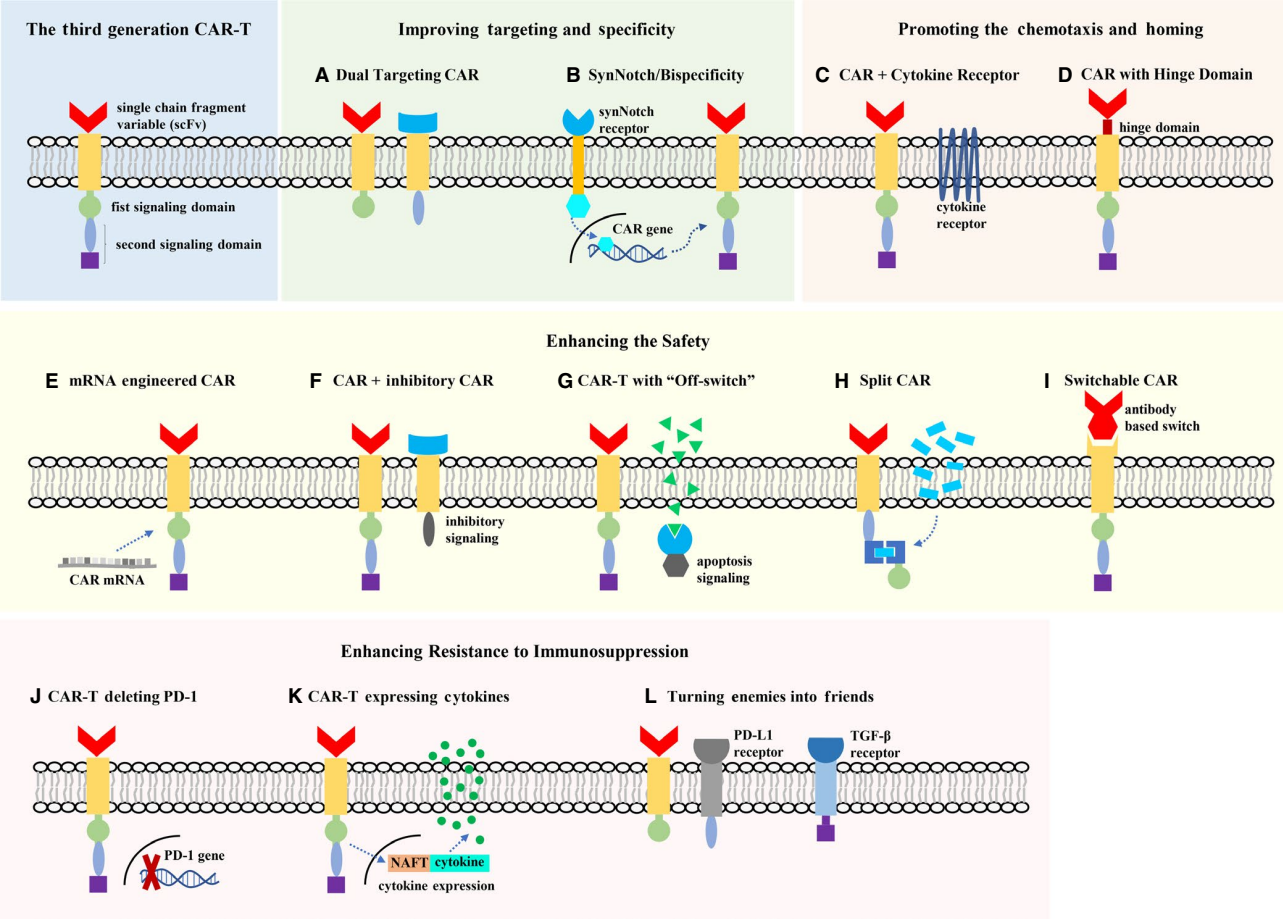
Design Optimization of CAR‐T Therapy. (A‐B) Targeting two tumor‐associated antigens could effectively improve the specificity of CAR‐T therapy. (C) Equipping CAR‐T with cytokine receptors or (D) incorporating a hinge domain in the CAR would promote the chemotaxis and homing of CAR‐T. Several strategies could enhance the safe application of CAR‐T, including (E) mRNA engineered CAR, (F) inhibitory CAR, (G) CAR‐T with “Off‐switch,” (H) the split CAR, and (I) the switchable CAR, which provide the opportunities to adjust the activation, function, and longevity of CAR‐T after its transfusion. (J) CAR‐T without PD‐1 expression avoided the immunosuppression of PD‐L1. (K) CAR‐T expressing additional cytokines, such as IL‐12 and IL‐18, would modulate the immunosuppressive microenvironment. (L) Recombination of the inhibitory immuno‐molecule receptors and activating signal domain would convert suppressive signals into activating signals, which also enhances the CAR‐T resistance to immunosuppression

Enhancing the specificity of CAR‐T therapy is the most effective way to improve the “on‐target, off‐tumor” effect, and this commonly utilizes a dual‐targeting CAR system. Zhang et al^39^ designed a CAR‐T system targeting both MSLN and CEA for PDAC. The CAR‐T cells expressed both MSLN‐CAR and CEA‐CAR, whose intracellular signaling domains, respectively, contained only the first or second signal required for T‐cell activation (Figure 1A). Thus, the interaction of such a dual‐targeted CAR‐T with either target alone would not effectively activate CAR‐T cells and perform antitumor effects, which would improve the specificity of CAR‐T treatment. Morsut et al^40^ used chimeric synNotch receptors within the CAR‐T system. The synNotch receptors were activated by targeting one tumor antigen, which activated the downstream Notch pathway to induce the expression of the CAR gene. The CAR specifically targeted another tumor antigen (Figure 1B). The CAR‐T system designed by Morsut is essentially another form of dual‐targeted CAR‐T that would also provide more design flexibility for regulating the CAR‐T function.

### 4.2 Promoting the chemotaxis and homing of CAR‐T Cells

The mismatch between chemokines and receptors, as mentioned above, influenced the recruitment of T lymphocytes in the microenvironment. Endowing CAR‐T cells with chemokine receptors that bound to tumor‐released chemokines would improve their homing ability (Figure 1C). In a study of CAR‐T therapy targeting GD2, which is expressed on neuroblastoma and malignant pleural mesothelioma cells, researchers observed that increasing CCR2 expression on the CAR‐T surface could enhance its tumor infiltration and antitumor capability.^41^, ^42^ Whilding's study^43^ also demonstrated that CXCR2‐expressing CAR‐T migrated more effectively toward IL‐8, which is secreted by multiple tumor cells, including pancreatic cancer cells. Due to the unique profiles of chemokine expression in different solid tumors, further study of chemokine expression would contribute to the design of new CAR‐T systems with improved tumor infiltration. In a study by Qin,^44^ incorporating a hinge domain between the scFv and the transmembrane region of the CAR could significantly increase tumor infiltration and proliferation after CAR‐T cell activation (Figure 1D), which might be explained by the membrane‐proximal location of the CAR‐recognized epitope, steric inhibitory effects between CAR and its target, or the high density of the target on tumor cells.

### 4.3 Enhancing the safety of CAR‐T

CAR‐T cells are usually generated by transferring the CAR‐expressing DNA sequence into T cells. Such CAR‐T cells continuously express the CAR and are activated when administered to patients, which makes it difficult to regulate their antitumor effect and inactivate them to reduce CAR‐T‐related side effects. Beatty et al^45^ designed a mRNA engineered CAR‐T using transcribed CAR‐encoding mRNA (Figure 1E). The CAR‐T cells in peripheral blood that could not bind to the solid tumor antigen would only transiently express CAR, which would improve the safety of CAR‐T therapy. Laboratory and clinical studies showed that the mRNA‐engineered CAR‐T not only had the same antineoplastic activity as traditional CAR‐T but also induced epitope spreading, which further promoted its antineoplastic effects. Adding an “inhibition switch signal” or an “apoptosis‐inducing switch” to the CAR‐T system is another effective way to ensure the safety of CAR‐T therapy. The former refers to introducing both CAR and inhibitory CAR (iCAR) into T cells (Figure 1F). The extracellular segment of iCAR targets antigen in normal tissues, while the intracellular segment of iCAR is composed of inhibitory signal sequence containing immunoreceptor tyrosine‐based inhibition motif from PD‐1 or CTLA‐4. When CAR‐T cells are activated by targeting TAA in normal tissues, iCAR can simultaneously provide an inhibitory signal to inhibit the CAR‐T activation, thus preventing the “on‐target, off‐tumor” effect to a certain extent.^46^ The latter is to introduce the caspase 9 apoptotic switch into CAR‐T cells. The switch was modified to recognize the small molecule compound ‘AP1903,’ which induced caspase 9 dimerization to initiate apoptosis and preferentially kill activated CAR‐T cells expressing high level of CAR (Figure 1G). In a phase I trial, it was found that more than 90% of CAR‐T cells could be removed within 30 minutes after ‘AP1903’ was administered.^47^ The design using apoptotic switch in CAR‐T is a kind of “off‐switch”. Accordingly, an “on‐switch” has also been devised to improve the safety of CAR‐T therapy. Wu et al^48^ designed a split CAR. The antigen binding component and the intracellular signaling component of the split CAR reassembled to form functional complexes only in the presence of a heterodimerizing small molecule, ‘Rapalog’ or ‘Gibberellin’ (Figure 1H). Split CAR‐T could only exert their antitumor effects when TAA and the heterodimerizing small molecule are simultaneously present, and the effect is similar to that of traditional CAR‐T. Raj et al^49^ devised a switchable CAR‐T system, which indirectly targeted tumor antigens but recognized a ‘switch’ containing both TAA‐binding Fab molecule and CAR‐binding domain (Figure 1I). Both of these designs incorporate molecular bridges in the process of CAR‐T activation, which allows physicians to control the timing, location, dosage, and activity of CAR‐T therapy by regulating the molecular bridge. Besides, when confronting the dilemma of antigen modulation, altering the ‘switch’ of the switchable CAR‐T would be cost‐effective.

### 4.4 Enhancing resistance to immunosuppression

The immunosuppression of CAR‐T is caused by immunosuppressive networks in tumor microenvironment. Rupp et al^50^ knocked out the PD‐1 gene in CAR‐T cells via CRISPR/Cas9 (Figure 1J). CAR‐T cells with PD‐1 deletion avoided the immunosuppression from PD‐L1 and showed stronger and more durable antineoplastic effects. In addition to inhibiting the negative immunoregulation of CAR‐T cells, enhancing positive immunoregulation can also increase the resistance to immunosuppression. T cells redirected for universal cytokine killing (TRUCK) endows CAR‐T cells with the ability to induce the secretion of specific cytokines.^51^ TRUCK‐CAR‐T cells were modified with an inducible cytokine expression cassette controled by the nuclear  factor of activated T cells (NFAT) promoter. The intracellular signal domain of CAR usually contains CD3ζ, which could induce NFAT activation and further activate the expression of transgenic cytokines (Figure 1K). Chmielewski et al^52^ designed a TRUCK‐CAR‐T system that expressed IL‐18 during CAR‐T activation. The TRUCK‐CAR‐T cells could not only induce an antitumor effect in PDAC and lung cancer models that are resistant to traditional CAR‐T therapy but also increase the number of M1 and NK cells and decrease the number of Treg, inhibitory DC, and M2 cells in the microenvironment, thus alleviating the microenvironmental immunosuppression. In addition to regulating the immune microenvironment and the immune status of CAR‐T cells, the extracellular domains of inhibitory cytokines such as PD‐1 and IL‐4 can be recombined with the intracellular domain that transmitted T‐cell activation signals to form new chimeric receptors, which “turns enemies into friends” and enables CAR‐T cells to be activated by inhibitory immune molecules and exert their antitumor effects^53^, ^54^ (Figure 1L).

## 5 FUTURE DIRECTIONS OF CAR‐T THERAPY FOR PDAC

In addition to the above optimization of CAR‐T design, a PDAC‐specific CAR‐T therapy should focus more on the unique profile of gene alterations and abundant stroma in PDAC. Besides, there may be CAR‐T subgroup with stronger antitumor effect which will be more effective against highly malignant PDAC. Thus, CAR‐T therapy for PDAC can be improved by the following directions.

### 5.1 Applying neoantigens as targets for CAR‐T therapy

The process of tumorigenesis involves the accumulation of gene mutations. Mutations in the tumor genome produce mutant proteins that are tumor specific, which are referred as neoantigens. Many attempts have been made to find new antigens and use them for immunotherapy in various cancer types,^55^ including PDAC.^55^ Neoantigens, which have high tumor specificity, exhibit distinctive advantages when used in CAR‐T therapy. In addition, tumors from different patients may express different neoantigens, and neoantigen‐guided CAR‐T therapy will conform better to the requirements of precision medicine. It is worthwhile to create novel methods with high sensitivity and specificity for seeking neoantigens associated with PDAC and to use them as targets for CAR‐T therapy.

### 5.2 Recognizing the most effective CAR‐T subgroup

During the preparation and transfusion of CAR‐T cells, no subgroups of CAR‐T cells were screened and eliminated. Heterogeneity may also exist in CAR‐T cell populations as well as in tumor cells. For instance, a study by Adusumilli et al^56^ showed that the therapeutic efficacy of MSLN‐CAR‐T therapy for lung cancer was dependent on early CD4+T cell activation and CD4+T cell‐mediated cytotoxicity, but not CD8+T cells. Therefore, we may find a CAR‐T subgroup with improved anticancer efficacy using specific methods, such as single‐cell sequencing or proteomics studies, and further test it in preclinical and clinical research.

### 5.3 Targeting abundant stroma of PDAC

Strong interstitial reaction is a distinct feature of PDAC that is not observed in other solid tumors. Up to 90% of the volume of PDAC tumors are composed of extracellular matrix (ECM).^57^ The activation of CAF is the primary source of ECM. CAF activation also produces abundant cytokines and tumor‐promoting proteins, which are closely related to PDAC progression.^58^ Therefore, designs of CAR‐T for PDAC should take the abundant stroma of PDAC into consideration. I. Identification of CAR‐T targets in PDAC stroma: CAF could be applied as a CAR‐T target for PDAC. Lo et al^59^ designed a CAR‐T therapy targeting fibroblast activation protein (FAP), which is a biomarker of tumor‐associated stromal cells, including CAF. In vivo, FAP‐CAR‐T cells effectively reduced the abundance of CAF, ECM content, and vascular density in PDAC stroma and restricted the growth of PDAC. Therefore, another biomarker of CAF or other stroma cells may also be used as targets of CAR‐T therapy. II. Utilization of CAR‐T as a drug‐delivering platform: Many targeted drugs have been developed according to the oncological mechanism of PDAC, including hedgehog pathway inhibitors, angiotensin inhibitors, hyaluronidase (PEGPH20), mTOR inhibitor, and PARP inhibitor.^58^, ^60^ The abundance of tumor stroma may prevent these drugs from accessing tumor tissues, which may weaken their antitumor effects. CAR‐T cells, which have good chemotaxis capability that can be modulated, can be used as a drug‐delivery platform to increase the drug concentration in the tumor microenvironment. Moreover, CAR‐T cells could be modified to directly express biologically active medicines, which would not only increase the concentration of drugs in PDAC but also facilitate combination therapy. III. Inspiration from non‐CAR‐T therapy: Strategies developed for non‐CAR‐T therapies might also be utilized for penetrating the interstitial barrier in CAR‐T therapy. For example, interstitial secreted protein, acidic and rich in cysteine, in PDAC has a high affinity for albumin, which enriches albumin‐bound paclitaxel in proximity to PDAC and enhances the delivery of paclitaxel into the tumor microenvironment.^61^ In a phase III study that enrolled patients with advanced PDAC, the combination of albumin‐bound paclitaxel and gemcitabine improved survival when compared with gemcitabine alone, while the addition of paclitaxel had no effect.^62^ Therefore, CAR‐T cells carrying albumin may acquire enhanced infiltrating and tumor‐killing capability.

## 6 CONCLUSION

CAR‐T therapy has great potential for killing tumors and has achieved great success in the treatment of hematological tumors. However, the use of CAR‐T therapy in solid tumors, such as PDAC, is still being explored. Challenges for the use of CAR‐T therapy for PDAC mainly include the immunosuppressive microenvironment, interstitial barrier, poor chemotaxis, and the “on‐target, off‐tumor” effect. Preclinical and early phase clinical studies of CAR‐T therapy targeting PDAC have shown encouraging results. However, more convincing clinical studies with larger sample sizes have yet to be conducted. Applying PDAC‐associated neoantigens as targets for CAR‐T therapy, recognizing the most effective CAR‐T subgroup for PDAC, and targeting abundant stroma of PDAC may contribute to develop a powerful CAR‐T therapy for PDAC in the future.

## CONFLICT OF INTEREST

The authors have declared no conflict of interest.
